# Mitochondrial quality control and communications with the nucleus are important in maintaining mitochondrial function and cell health^☆^^☆☆^

**DOI:** 10.1016/j.bbagen.2013.10.041

**Published:** 2014-04

**Authors:** Vassilios N. Kotiadis, Michael R. Duchen, Laura D. Osellame

**Affiliations:** a Department of Cell and Developmental Biology, University College London, WC1E 6BT, UK; b UCL Consortium for Mitochondrial Research, University College London, WC1E 6BT, UK

**Keywords:** Mitochondria, Quality control, Antioxidant defence, Mitophagy, Retrograde signalling, Protein homeostasis

## Abstract

**Background:**

The maintenance of cell metabolism and homeostasis is a fundamental characteristic of living organisms. In eukaryotes, mitochondria are the cornerstone of these life supporting processes, playing leading roles in a host of core cellular functions, including energy transduction, metabolic and calcium signalling, and supporting roles in a number of biosynthetic pathways. The possession of a discrete mitochondrial genome dictates that the maintenance of mitochondrial ‘fitness’ requires quality control mechanisms which involve close communication with the nucleus.

**Scope of review:**

This review explores the synergistic mechanisms that control mitochondrial quality and function and ensure cellular bioenergetic homeostasis. These include antioxidant defence mechanisms that protect against oxidative damage caused by reactive oxygen species, while regulating signals transduced through such free radicals. Protein homeostasis controls import, folding, and degradation of proteins underpinned by mechanisms that regulate bioenergetic capacity through the mitochondrial unfolded protein response. Autophagic machinery is recruited for mitochondrial turnover through the process of mitophagy. Mitochondria also communicate with the nucleus to exact specific transcriptional responses through retrograde signalling pathways.

**Major conclusions:**

The outcome of mitochondrial quality control is not only reliant on the efficient operation of the core homeostatic mechanisms but also in the effective interaction of mitochondria with other cellular components, namely the nucleus.

**General significance:**

Understanding mitochondrial quality control and the interactions between the organelle and the nucleus will be crucial in developing therapies for the plethora of diseases in which the pathophysiology is determined by mitochondrial dysfunction. This article is part of a Special Issue entitled Frontiers of Mitochondrial Research.

## Highlights

• Mitochondria are critical components of the eukaryotic cell, responsible for diverse ranges of functions.
• While ROS are damaging in excess amounts, low levels are essential signals in homeostasis.
• Mitophagy is a highly regulated form of autophagy regulated by PINK1 and Parkin.
• Transcription factors activate and coordinate the expression of both nuclear and mitochondrial genes.

## 1 Introduction — mitochondrial function and fitness are fundamental to cellular health

Mitochondria are critical cellular organelles best known for their role in providing efficient energetic support through the chemiosmotic process of oxidative phosphorylation (OXPHOS). Although mitochondria were first observed in the latter part of the 19th century, their role in aerobic energy transduction through the characteristic chemiosmotic mechanism of OXPHOS first began to be clarified in the 1960s [1]. Since then, mitochondria have also been shown to perform a variety of roles in processes such as the transduction of metabolic and stress signals [2,3], the production of free radicals such as reactive oxygen species (ROS) [4], and the induction of programmed cell death [3]. Central to cellular bioenergetic homeostasis is the requirement to sustain the mitochondrial population to ensure that cellular energy demands are met by energy supply. A variety of quality control (QC) mechanisms have evolved which ensure cellular bioenergetic homeostasis, managing mitochondrial components, products and by-products. Dysregulation of these pathways is emerging as a key theme in understanding many contemporary human diseases. Thus, it is increasingly clear that mitochondrial dysfunction and disordered regulation of mitochondrial homeostasis play integral roles in the pathophysiology of conditions such as cancer [5], neurodegeneration [6], as well as ageing [7]. In addition, dysfunction of mitochondria caused by mutations in genes coding for the organelle components typifies a distinctive class of conditions known as mitochondrial diseases [8].

Mitochondrial structure is characterised by two protein containing lipid bilayers. The main compartments of the organelle are the external outer mitochondrial membrane (OMM), the inter membrane space (IMS) and the matrix, which is enclosed by the inner mitochondrial membrane (IMM) that undergoes intense folding into cristae, which vastly expand the membrane surface area. The electron transport chain (ETC.) complexes work together through cellular respiration to generate the proton gradient across the IMM that delivers the crucial chemiosmotic force of the OXPHOS process. The biochemistry that underpins the OXPHOS process revolves around a variety of redox reactions, the transfer of electrons between protein complexes, and the eventual delivery of electrons to molecular oxygen (O_2_). However, stray electrons can react prematurely and incompletely with O_2_ in the local environment, especially if the respiratory chain is compromised, leading to the formation of free radicals known as reactive oxygen species (ROS) [4,9]. Primarily considered dangerous by-products that can impair the structure and activity of proteins and lipids, ROS also appear to be essential signalling components of cellular physiology [10,11]. Nevertheless, production of ROS can lead to the accumulation of damaged mitochondrial components. Primary cellular defences against this type of oxidative stress have been resourcefully adapted so as to detoxify the harmful effects of ROS while maintaining their signalling capacity.

Loss of activity, misfolding, and aggregation of protein components due to genetic mutations or environmental stress are important causes of mitochondrial dysfunction that lead to deterioration in cellular health [12–14]. The replenishment of mitochondrial proteins is a complex process, as it requires the coordinated expression of two genomes, the nuclear and mitochondrial. The human mitochondrial genome encodes only 13 proteins, all of which are critical components of the respiratory chain complexes, as well as two ribosomal subunits and all the tRNAs required for protein translation [15]. All other mitochondrial proteins are encoded by the nuclear genome. Formation of the functional OXPHOS complexes is heavily reliant on a strict stoichiometry of protein components in order to correctly assemble in the IMM [16]. Hence, the effective and measured transcription and translation of both genomes must be coordinated to avoid superfluous expression and accumulation of non-functional proteins within mitochondria. The nuclear genome also encodes for mitochondrial targeted proteins responsible for regulating mtDNA encoded gene expression and replication, thus playing a major part in regulating mitochondrial genome function and capacity [17]. This pathway is complemented by the emerging role of the converse regulation of nuclear gene expression in response to cues originating from the mitochondria, in a process termed retrograde signalling [18,19]. Hence, mechanisms of mitochondrial QC include the monitoring and control of components, function, and products, as well as the communication between mitochondria and other cellular components. During this review we aim to highlight current understanding of mitochondrial QC processes as they relate to different aspects of mitochondrial structure and function, as well as remarking on various associated systems that are essential for QC.

## 2 Antioxidant defences and the regulation of reactive oxygen species (ROS)

### 2.1 The multifaceted ROS — oxidative stress and physiological requirement for ROS

Oxidative stress is an environmental condition where ROS generated exceeds the capacity of antioxidant defences in neutralising free radicals, and thus results in damage caused by the aberrant reactivity of ROS with cellular components. Oxygen derived free radicals are an important by-product of the mitochondrial ETC., not only because of their potential to cause damage, but also because they are thought to play important signalling roles essential for physiological cellular function [9,11]. ROS are initially formed by the premature release of electrons from the ETC. and the reduction of molecular oxygen (O_2_) to form the superoxide radical (^•^O_2_^−^) [4,9]. Superoxide is thought to be generated at seven different sites associated with proteins at the IMM, which mainly generate superoxide on the matrix side of the IMM. Of these sites, two also release O_2_^−^ into the IMS [10]. Superoxide has a very short half-life and is highly reactive, causing damage to vital mitochondrial components such as mtDNA, lipid membranes and to respiratory complexes, all of which are located near the sites of superoxide production [9,10]. Superoxide can then be transformed into other free radical species via enzymatic catalysis mediated by superoxide dismutases that generate hydrogen peroxide (H_2_O_2_), by iron (Fe) mediated chemistry forming the highly reactive hydroxyl radical (^•^OH^−^), and also reacting with nitric oxide (^•^NO) to form the highly reactive and very damaging peroxynitrite anion (^•^ONOO^−^).

**Fig. 1:**
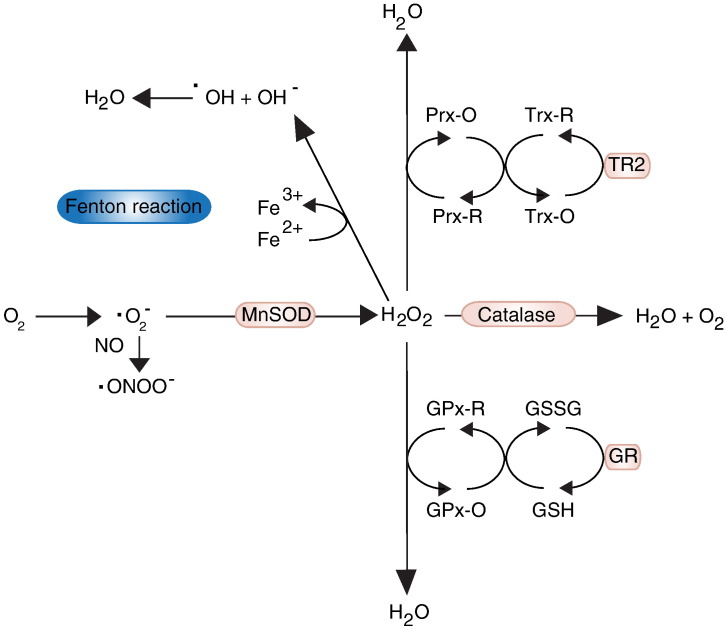
The path of ROS. Superoxide radicals (^•^O_2_) are typically converted to hydrogen peroxide (H_2_O_2_) by mitochondrial manganese superoxide dismutase (MnSOD). H_2_O_2_ can readily diffuse through lipid membranes and react with a multitude of antioxidant components. At low concentrations H_2_O_2_ is most frequently converted to H_2_O through the activity of peroxiredoxins (Prx-O: oxidised; Prx-R: reduced), but at higher concentrations glutathione peroxidases (GPx-O: oxidised; GPx-R: reduced) can provide additional support in facilitating this conversion in order to deal with oxidative stress. Once oxidised by H_2_O_2_, both Prx and GPx can transfer their redox state to thioredoxins (Trx-O: oxidised; Trx-R: reduced) and glutathione (GSSG: oxidised; GSH: reduced) respectively and are thus recycled back to a reduced state. Reduction of Trx and GSH is catalysed by thioredoxin reductase (TR2) and glutathione reductase (GR) respectively. Catalase converts H_2_O_2_ to H_2_O but due to its peroxisomal location is typically functional only under increased oxidative stress conditions. When there is a build of ^•^O_2_^−^ and H_2_O_2_ these can react with iron (Fe) according to Fenton chemistry, and generate the highly reactive and damaging hydroxyl radical (^•^OH^−^). If ^•^O_2_^−^ is not dealt with sufficiently by MnSOD it can react with ^•^NO and generate the peroxynitrite radical (^•^ONOO).

The presence of Fe in an environment in which H_2_O_2_ has been released can lead to the generation of ^•^OH^−^ on the basis of Fenton chemistry [20] (Fig. 1). The process is comprised of the oxidation of ferrous iron (Fe^2 +^) in the presence of H_2_O_2_ to form ferric iron (Fe^3 +^), which concomitantly releases a hydroxyl radical (^•^OH^−^), followed by the reduction of the iron back to Fe^2 +^ by the same H_2_O_2_ molecule that also releases a proton and a hydroperoxyl radical (^•^OOH^−^) (i.e. a protonated form of ^•^O_2_^−^). The following reaction describes the Fenton chemistry involved in ^•^OH^−^ production from H_2_O_2_ reacting with Fe^2 +^:

Fe^2 +^ + H_2_O_2_ + H2 → Fe^3 +^ + ^•^OH^−^ + H_2_O

Fe^3 +^ + H_2_O_2_ → Fe^2 +^ + ^•^OOH^−^ + H^+^.

In order to prevent oxidative stress generated through Fenton chemistry, cells have evolved the capacity to regulate iron availability throughout the cell through specific storage components known as ferritins, which are able to regulate the release of iron within specific compartments such as the mitochondria [21]. Another protein linked with iron homeostasis in mitochondria is frataxin, which is primarily thought to be involved in facilitating iron–sulphur (Fe–S) cluster biogenesis. Mutations that lead to the reduced expression of frataxin are strongly linked with the neurodegenerative disease Friedreich's ataxia [22].

^•^O_2_^−^ also readily reacts with ^•^NO resulting in the formation of ^•^ONOO^−^, a free radical species which can have a major effect on cellular function and health [23]. ^•^ONOO^−^ and its products can potentially have a multitude of effects on protein, lipid, and DNA components, either through oxidation or nitration. For instance, the essential co-factor tetrahydrobiopterin (BH_4_), of the endothelial nitric oxide synthase (eNOS), is highly sensitive to oxidation by ^•^ONOO^−^. Loss of BH_4_ function through oxidative damage can lead the eNOS to generate ^•^O_2_^−^ and contribute further to oxidative stress. This cycle of damage to eNOS by ^•^ONOO^−^ is thought to be an important pathophysiological factor in vascular disease [24]. The reaction of ^•^ONOO^−^ with other molecules can also lead to the formation of more damaging ROS. An example of this activity of ^•^ONOO^−^ is the reaction with CO_2_ in the aqueous phase leading to the formation of carbonate (^•^CO_3_^−^) and nitrogen dioxide (^•^NO_2_) [25]. Both ^•^CO_3_^−^ and ^•^NO_2_ are potent one electron oxidants and are thought to readily cause damage to proteins, lipids, and DNA [23]. It is clear that the cellular elements exposed to superoxide in the immediate environment of its generation will determine the downstream consequences of ROS generation.

In contrast to the damaging effects of excessive ROS production, low levels of ROS produced by mitochondria appear to operate as important signals involved in maintaining cellular homeostasis and inducing stress responses [4]. As primary components involved in oxygen utilisation, mitochondria can also act as oxygen sensors and induce appropriate responses during hypoxic conditions. Interestingly, the mechanism that senses low levels of oxygen is thought to typically require the release of H_2_O_2_ by mitochondria in order to activate the transcriptional regulator known as hypoxia induced factor 1α (HIF-1) [11]. Although the HIF-1 subunit HIF-1α is constitutively expressed, it is hydroxylated by prolyl hydroxylase (PHD) on proline residues and marked for rapid degradation during normoxia. However, H_2_O_2_ appears to inhibit this activity of PHD and thus leads to stabilisation of HIF-1α, which activates the transcription of factors involved in hypoxic response [9]. Although the generation of ROS (which is highly reliant on oxygen availability) may seem paradoxical as a signal under hypoxic conditions, it may form part of an effective system of mitochondrial oxygen sensing. Nevertheless, there is some controversy surrounding this subject and in various models of hypoxic pulmonary vasoconstriction there is evidence to support both the increase and decrease in ROS as signals for low oxygen sensing [26]. Furthermore, the generation of H_2_O_2_ by mitochondria has also been shown to be essential for autophagic mechanisms during starvation conditions, as H_2_O_2_ release by mitochondria appears to play an important role in the induction of autophagosome formation [27]. In addition to these physiological roles, low level ROS release by mitochondria has also been linked to the activation of stress response regulators such as NF-kappa B (NFκB), JNK-1, and p53 [28–30]. In order to effectively deal with the contrasting effects of high and low ROS concentrations on mitochondrial and cellular function ROS are targeted by a variety of components that make up antioxidant defences.

### 2.2 ROS scavengers — the first line of defence

Electrons released by the ETC. react with molecular oxygen (O_2_) forming the superoxide radical (^•^O_2_^−^), which is rapidly converted to H_2_O_2_ within mitochondria by the mitochondrial manganese superoxide dismutase (MnSOD), also known as SOD2 (Fig. 1). Although no mutations in SOD2 have been directly linked to a disease state, mutations in the SOD1 gene coding for its cytoplasmic counterpart, the copper–zinc superoxide dismutase (CuZnSOD), are associated with familial Amyotrophic Lateral Sclerosis (ALS), through a toxic gain of function effect [31]. An important role of SOD is its effective dismutation of superoxide in order to prevent its reaction with nitric oxide that leads to the formation of the highly deleterious peroxynitrite species [23].

Once ^•^O_2_^−^ is converted to H_2_O_2_ it can be sequestered and transformed by peroxiredoxins (Prxs), glutathione peroxidases (GPxs), or catalase. In mammalian cells there are two Prx isoforms targeted to mitochondria, both working through the oxidation of an active cysteine site by H_2_O_2_ (Fig. 1) [32]. Subsequent reduction of the active sites by thioredoxin (Trx) can reverse the effects of H_2_O_2_ and allow recycling of Prxs. Similarly, Trx can also be recycled and kept in a reduced state through an NADPH dependent reaction with the flavoenzyme thioredoxin reductase. The ability of Prxs to transfer the oxidation state to other proteins is thought to be part of a H_2_O_2_ sensing mechanism that contributes to various redox signalling pathways that may modulate mitochondrial processes such as the TCA cycle, haem biosynthesis, and OXPHOS in response to environmental changes [32,33]. There are eight GPxs found within mammalian cells that can be oxidised by H_2_O_2_ and consequently reduced by glutathione (GSH) [34].

Interestingly, there is always the danger that glutathione capacity can be depleted by certain types of ROS, particularly ^•^ONOO^−^ and its products [35]. This may be a major factor in the progression of pathophysiology characterised by increased ROS leading to oxidative stress, and highlights the importance of the GPx system in redox regulation [23]. Similarly to Prxs, the capacity of GPxs to transfer their oxidation state to other proteins, in this case through the S-glutathionylation of cysteine residues on the recipient proteins, is thought to be part of signalling systems involved in the regulation of the mitochondrial redox state [34]. Both Prxs and GPxs can function as buffers that limit H_2_O_2_ induced oxidative stress, but can also function in concomitant roles as part of signalling pathways that utilise ROS and lead to the modulation of metabolic and biosynthetic mitochondrial processes [32,33]. Moreover, the relative abundance of Prxs and GPxs within mitochondria provides a clue on their differing roles in detoxifying ROS. The high activity and abundance of Prx are thought to be important for converting low levels of ROS, and as such are more likely to act as the final receptor of low levels of ROS associated with signalling [4]. Although GPxs have a similar activity to Prxs, they are far less abundant and cannot compete in substrate sequestration at low levels of H_2_O_2_ [33]. While Prxs would be most beneficial under physiological conditions where low levels of ROS must be accurately coordinated; GPxs can provide the added support required for detoxifying the radicals in higher concentrations.

Other cellular defences against mitochondrial derived ROS include catalase, which is another enzyme that can detoxify H_2_O_2_. Catalase converts H_2_O_2_ into H_2_O and O_2_ and is typically found in peroxisomes (Fig. 1). Although not directly involved in mitochondrial function, it provides defence against oxidative stress that can be relevant to mitochondria, as demonstrated by conditions where mitochondrial function is affected by insufficient or aberrant catalase functionality. Genetic abnormalities in catalase levels and function have been shown to be associated with the onset of type II diabetes, suggesting an impact of the activity of catalase upon insulin signalling [36].

Ubiquinone, or coenzyme Q (CoQ), is a vitamin like lipid soluble molecule that features prominently in most cellular lipid membranes, but is also found in substantial amounts in the IMM [37]. At the IMM, CoQ functions as part of the ETC. where it facilitates transfer of electrons from complexes I and II to complex III by undergoing a triphasic redox cycle, known as the Q cycle, which allows it to exist in a completely reduced (ubiquinone) or oxidised (ubiquinol) form. A concurrent role of ubiquinone is its involvement in superoxide formation through the Q cycle intermediate known as ubisemiquinone, which binds at the Qi and Qo sites on complex III [9,10]. Although formation of ROS at complex III in this manner is thought to have a physiological role in the HIF-1 oxygen sensing mechanism, under most conditions it is thought to be overshadowed by the ROS producing capacity of complex I [9]. Conversely, this redox cycling capacity also allows CoQ to function in an antioxidant role where amongst other protective effects it can alleviate lipid peroxidation [38]. Adding to the importance of CoQ in combating mitochondrial ROS is the recent development of an exogenously derived mitochondria targeted CoQ analogue known as MitoQ_10_, with in-vitro studies demonstrating that it can alleviate a variety of aberrant phenotypes associated with increased mitochondrial ROS [34,38–41]. The capacity of CoQ to efficiently function as an antioxidant and the positive effects that follow suggest that its abundance in the IMM may have more purpose than just to function as an electron carrier in the ETC. A major consequence of the production of damaging ROS at mitochondria is the peroxidation of lipids, in particular at the IMM which contains a large amount of unsaturated fatty acids [42–45]. Such damage can lead to a considerable deterioration in OXPHOS and other functions of mitochondria that rely on lipid membrane composition and integrity. As such, it is also important to acknowledge the capacity of CoQ and the IMM as an important facilitator of antioxidant responses, and thus an important component of mitochondrial QC.

Damage caused by oxidative stress to cellular components such as proteins and lipids can lead to the activation of stress response pathways. An interesting example of such a system is the Kelch-like ECH-associated protein 1 (Keap1)–NF-E2-related factor 2 (Nrf2) signalling pathway that increases the expression of cytoprotective genes in response to oxidative stress [46]. Nrf2 acts as a transcriptional activator through its Cap'n'collar (CNC) leucine zipper DNA binding region and interaction with small Maf proteins [47]. However, Nrf2 is kept in an inactive state, bound to Keap1, and the complex is readily ubiquitinated and degraded. A variety of oxidative stress signals, including oxidised fatty acids, react with Keap1 inducing a conformational change that will allow the dissociation and activation of Nrf2 [46]. All of these antioxidant systems are a first line of defence against the harmful effects of ROS, but also appear to act as regulators of their signalling activity. In both capacities they are extremely important in controlling the quality of mitochondrial function and maintaining homeostasis and cellular health.

## 3 Mitochondrial component control and protein homeostasis

### 3.1 Uptake and folding

Mitochondrial proteins not encoded by mtDNA are imported as unfolded polypeptides through a highly selective mechanism [48]. Proteins that contain a mitochondrial targeting sequence (MTS) are targeted to mitochondria where they are imported by the organelle in a mitochondrial membrane potential (ΔΨ_m_) dependent manner that requires the activity of the outer and inner membrane translocators (TOM & TIM respectively) [49]. The concerted action of TOM, TIM, with the aid of chaperones and proteases, facilitates the translocation of the pre-proteins, cleavage of the MTS, and folding of the proteins to their native form within mitochondria [48]. The matrix localised Hsp60 chaperonin, formed by Hsp60 and Hsp10 subunits, directs the transport and folding and regulates the appropriate localisation of small soluble proteins imported to mitochondria [50,51]. Hsp70 is another matrix localised chaperone involved in protein import, folding and complex assembly but also has a significant role in iron–sulphur cluster formation [52,53]. Both Hsp60 and Hsp70 are important in protein folding and ETC. complex assembly, while they also act in preventing the aggregation of unfolded or misfolded proteins [54].

### 3.2 Degradation and clearance directing

Proteins that fail to fold or assemble in the matrix or IMS are typically dealt with by an array of mitochondrial proteases. ClpXP and Lon are matrix localised AAA proteases responsible for degrading soluble proteins [55,56]. Lon has been shown to preferentially degrade certain oxidised proteins such as aconitase [57]. Furthermore, the matrix localised m-AAA and IMS localised i-AAA proteases are primarily tasked with degrading any misassembled or unfolded protein OXPHOS components [58]. Mutations in the gene coding for paraplegin, a main component of the m-AAA protease, cause the debilitating neurodegenerative disease spastic paraplegia, highlighting the importance of the protease's function in maintaining cellular health [59]. In addition, the m-AAA protease has been shown to influence the expression of mtDNA encoded subunits by controlling the processing of the nuclear encoded mitochondrial ribosomal protein MrpL32 during import [60].

Interestingly, recent findings show that during conditions of oxidative stress there is inhibition of the processing of MrpL32 by the m-AAA protease, which leads to a decrease in protein translation within mitochondria [61]. The importance of maintaining protein populations within mitochondria in the correct form is highlighted by the many diseases associated with the loss of protein QC in the organelle [62,63]. Although the clearance of dysfunctional proteins within mitochondria is usually accomplished efficiently by the mitochondrial proteases, the OMM proteins require external support. Due to the position of the OMM in fronting the mitochondrial boundary with the cytoplasm, proteins facing the cytoplasm can be processed by the cytoplasmic ubiquitin proteasome system (UPS) that specifically targets and degrades ubiquitin tagged proteins [64]. Several mitochondrial E3 ubiquitin ligases found in the OMM are known to play a role in divergent mechanisms such as mitochondrial dynamics, stress response, and apoptosis [65–68]. These include resident OMM proteins such as MARCH V/MITOL [66,69,70], MULAN/MAPL [71,72] and RNF185 [73]. Involved in sensing mitochondrial dysfunction, the cytosolic E3 ubiquitin ligase Parkin, translocates to the OMM and is responsible for pan ubiquitination of OMM proteins such as components of the TOM complex (20, 40 and 70), Mfn1/2, Miro1/2 and Fis1 [74] (further described in Section 4.2). Furthermore, a plethora of ubiquitinated mitochondrial proteins have been identified through proteomic studies, suggesting that the UPS is a commonly deployed degradation method for the QC of mitochondrial proteins [75].

### 3.3 Coping with change — the mitochondrial unfolded protein response (UPR^mt^)

**Fig. 2:**
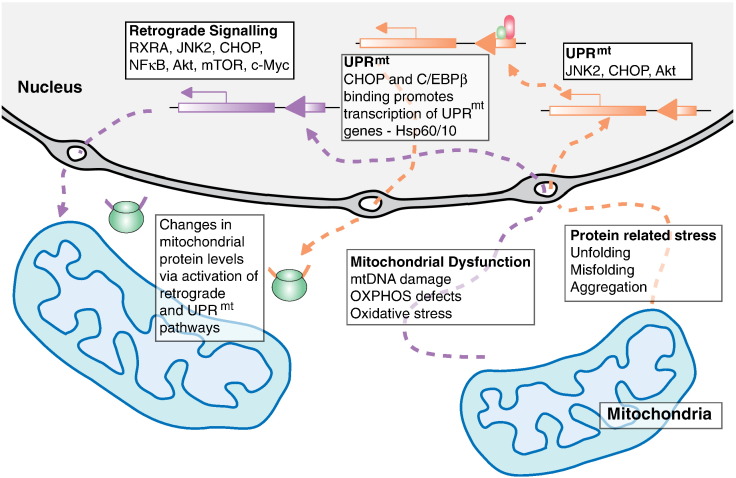
Factors involved in mitochondrial retrograde signalling and UPR^mt^. Mitochondria-nuclear crosstalk is activated under mitochondrial stress. The signalling pathways activated during mammalian retrograde responses and mitochondrial unfolded protein response (UPR^mt^) have not yet been clarified, yet the activation of certain signalling and transcription factors has been observed. The UPR^mt^ is activated by accumulation of unfolded, misfolded or aggregated proteins in the mitochondrial matrix. This is a two-step process that usually involves activation of transcription factors including CCAAT-enhancer-binding protein homologous protein (CHOP). CHOP dimerises with CCAAT-enhancer-binding protein β (C/EBPβ) and binds to and promotes transcription of UPR^mt^ responsive genes containing a CHOP-C/EBPβ element. This leads to transcription of mitochondrial quality control genes including Hsp60/10. Reterograde signalling is activated by mitochondrial dysfunction including mtDNA/OXPHOS defects and oxidative stress. This triggers activation of cytosolic signalling pathways which lead to activation of transcription factors including RXRA, JNK2, CHOP, NKκB, Akt, mTOR and c-Myc and transcription of nuclear genes that produce adaptive changes in mitochondrial protein levels.

Although mitochondrial chaperones and proteases can typically deal with the bulk of import, folding, and degradation of proteins within mitochondria, there are instances when this capacity can be overwhelmed by demand. Increases in unfolded proteins and unassembled complexes within mitochondria can come about either through genetic mutations that may alter protein sequences or through environmental conditions that may alter protein structure. As a response to this, the mitochondrial protein homeostasis machinery appears to be able to induce a signal towards the nucleus in order to increase its capacity through the mitochondrial unfolded protein response (UPR^mt^) [54,76]. The UPR^mt^ appears to be similar to another eukaryotic stress response mechanism, the endoplasmic reticulum unfolded protein response (UPR^ER^). Characterisation of the UPR^ER^ in mammalian models has been more successful than its mitochondrial counterpart, yet the general theme of a cellular compartment sensing unfolded protein stress, transmitting a signal to the nucleus, and the resulting upregulation of organelle specific chaperones and proteases reflects and provides a precedent for the UPR^mt^ [54]. Furthermore, downstream components utilised by the UPR^ER^, such as the transcription factor CCAAT-enhancer-binding protein homologous protein (CHOP), have also been shown to be involved in UPR^mt^ and provide a mechanistic link between the two systems (Fig. 2). The UPR^mt^ system has been best characterised in the nematode worm model *Caenorhabditis elegans*, but there is substantial work supporting the existence of this system in mammalian cells. Key discoveries on the mammalian UPR^mt^ were made in the Hoogenraad lab where it was demonstrated that disruption in the expression of mitochondrial proteins led to the upregulation of specific mitochondrial chaperones and proteases, such as Hsp60 and ClpP respectively [77,78]. Further work demonstrated the involvement of the transcription factor CHOP, which appears to be upregulated through Jun signalling components, when unfolded proteins accumulate in the matrix [78,79]. Although intermediate components of this system in mammalian cells are still largely unknown, several insights have been gained by the elucidation of the mechanism in *C. elegans*. Within this model, it is understood that unfolded proteins accumulating within mitochondria are degraded to small peptides by the protease ClpP in the matrix, which are then transported across the IMM by HAF-1 where they diffuse across the OMM and into the cytosol [54]. The increased concentration of these peptides leads to the activation of the bZip transcription factor ATFS-1 that accumulates within the nucleus [80]. Furthermore, the UBL-5 and DVE-1 transcriptional co-factors accumulate within the nucleus due to HAF-1 mediated peptide efflux from the mitochondrial matrix. These interact with ATFS-1 and induce the expression of mitochondrial chaperones such as Hsp60 and Hsp70 [80]. The UPR^mt^ is an interesting example of the interaction required between mitochondria and the nucleus in order to mediate an appropriate response to stress generated by abnormalities to fundamental mitochondrial components, in this case proteins.

## 4 Controlling the mitochondrial cohort through elimination — autophagy

### 4.1 Autophagy — a two faced process

**Fig. 3:**
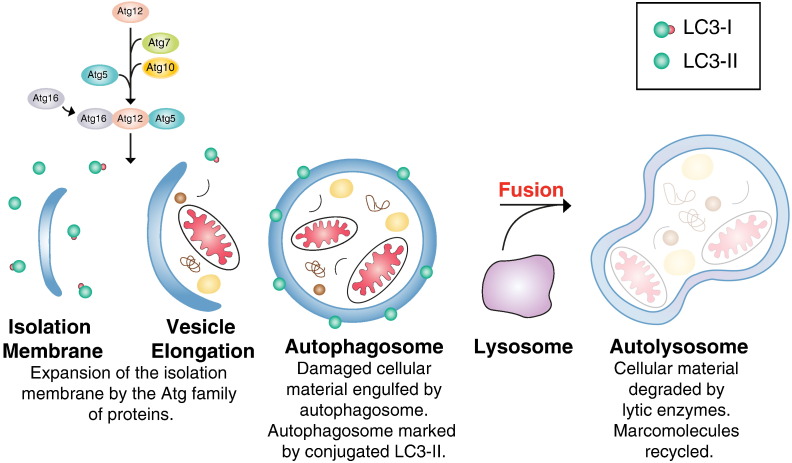
The autophagic pathway. The isolation membrane is formed in the cytosol and expanded by the conjugation of the Atg family of proteins (Atg proteins 12,7,10 and conjugated Atg 5/12/16). LC3 exists as both LC3-I and LC3-II. (Damaged organelles and proteins with a long half-life are engulfed by the newly formed autophagosome. The double membrane structure is marked by LC3-II (conjugated to phosphatidylethanolamine) on the surface. The autophagosome fuses with the lysosome under low pH conditions to form the lytic vesicle the autolysosome. Within this compartment organelles and proteins are degraded by lysosomal hydrolyses. Schematic not drawn to scale.

Macroautophagy (hereon referred to as autophagy), the cellular lysosomal degradation pathway, is a catabolic process that is essential for homeostasis, development and survival [81]. Autophagy occurs in almost all cells and performs a regulatory role in removing redundant/damaged organelles and proteins. It can be up or down-regulated in response to nutrient starvation or high metabolic demand and appears to be downregulated in several disease states [82–84]. In addition to the lysosome, the key component of this pathway is the autophagosome. Nucleation and elongation of the autophagosome are reliant on several autophagy-related (Atg) proteins that partake in ubiquitylation-like reactions. Atg5-12 and Atg16L are involved in the elongation step of the process while microtubule-associated protein 1 light chain (LC3; also known as Atg8) is present in two forms; LC-I and LC3-II depending on the progression of the pathway (Fig. 3) [85]. In its cytosolic form it is known as LC3-I, however when proteolytically processed and covalently conjugated to phosphatidylethanolamine on the autophagosomal membrane it is known as LC3-II [85]. The conjugation marks the maturation of the autophagosome, which engulfs damaged organelles and proteins (long half-life). The fully formed double membrane organelle fuses with the lysosome to form the autolysosome (Fig. 3). The combination of low pH and lytic enzymes within the autolysosome degrade organelles and proteins sequestered by the autophagosome (the inner membrane of the autolysosome is also degraded in this process). The resulting macromolecules can be recycled for reuse during starvation.

Although autophagy is a general term for turnover of bulk cytosolic components, different terms are used to describe the selectivity of the process depending on the organelle; the endoplasmic reticulum (ER; reticulophagy) [86,87], peroxisomes (pexophagy) [88], ribosomes (ribophagy) [89] and mitochondria (mitophagy) [90].

### 4.2 Mitophagy — a highly selective form of autophagy

Although mitochondria can be non-selectively degraded by macroautophagy, both yeast and mammalian cells have the ability to selectively turnover damaged mitochondria though a process called mitophagy; a more tightly regulated process than that of the macroautophagy pathway [90,91]. Mitophagy limits activation of the intrinsic apoptotic pathway by targeting and removing dysfunctional mitochondria prior to permeabilisation of the outer membrane. The initiation of this pathway is regulated at organelle level, thus allowing for removal of a single mitochondrion. The specificity of the pathway was first demonstrated by using a proton ionophore (CCCP) to uncouple the respiratory chain or photo-irradiation to depolarize individual mitochondria [92,93], inducing autophagic clearance of mitochondria while leaving other organelles intact. Induction and regulation of mitophagy is mechanistically separate from macroautophagy and involves co-ordination of the mitochondrial morphology machinery (in particular fission), PINK1 and Parkin and various effector proteins.

### 4.3 Regulation of mitochondrial morphology — a requisite step in mitophagy

Mitochondria are dynamic organelles whose shape and size are governed by the opposing processes of fission and fusion, operating concurrently with organelle trafficking and distribution mainly along microtubules in mammalian cells and along the actin cytoskeleton in yeast. This balance maintains and controls the inheritance and transmission of mtDNA, metabolite levels and energy transduction [94]. Mitochondrial morphology is regulated by a conserved family of large GTPases in both the inner and outer membranes [95]. Optic atrophy 1 (Opa1, Mgm1 in yeast) maintains and controls inner membrane fusion and cristae integrity, while fusion of the outer membrane is largely under control of the two mitofusins (Mfn1 and Mfn2, Fzo1 in yeast) [96,97]. Like fusion, fission is also controlled by a large GTPase, dynamin related protein 1 (Drp1/Dlp1, Dnm1p in yeast). Drp1 is a largely cytosolic protein that cycles on and off the mitochondrial outer membrane, only present on the mitochondrial surface in multimeric structures when required for scission of the organelle [98]. Although the exact mechanism of Drp1 recruitment to the outer membrane remains elusive, new receptor/adaptor molecules have been proposed (Mff and MiD49/51), however further mechanistic clarification is required to ascertain how these proteins mediate this process [99,100].

Modulation of mitochondrial shape is one of many important steps in mitophagy and has been proven by manipulation of pro-fission proteins Fis1 and Drp1 and appearance of fragmented mitochondria prior to mitophagy [101]. Knockdown of Fis1 or overexpression of a dominant negative form of Drp1 (Drp1^K38A^) results in almost complete fusion of the mitochondrial network [101], reducing mitochondrial sequestration by autophagosomes. In keeping with this, many studies have linked the rate of mitophagy to the size of mitochondria. Mitochondria visualized within autophagosomes have been reported to be < 1 μm in diameter [101–104]. However this is not seen uniformly, making it complex to delineate. Both, overexpression of Opa1 (resulting in a fragmented network) and inhibition of fission (a tubular, extended network) in INS1 cells, result in a ~ 70% reduction in mitophagy rates [101]. These conflicting results suggest that organelle length may not be a rate-limiting step in mitophagy.

What is clear however, is the involvement of both division and fusion of mitochondria leading up to mitophagy. In contrast to fission, fusion requires an intact membrane potential (ΔΨ_m_) and is thus selective. Termed ‘kiss-and-run’ events these are brief fusion events followed by fission of the organelle. These events often result in daughter mitochondria of unequal ΔΨ_m_ [101]. This single event is enough to trigger induction of mitophagy that degrades the daughter organelle with a lower potential. However, if the mitochondrion can recover its ΔΨ_m_, it can re-enter the fission/fusion cycle [101]. This suggests that mitochondrial fission, which is often a cellular response to stress, is a mechanism utilised by the cell to segregate and eliminate damaged mitochondria from an otherwise healthy network.

### 4.4 PINK1 and Parkin

Mutations in PTEN-induced kinase (PINK1) and Parkin can cause familial Parkinson's disease [105,106]. These proteins are key regulators in mitophagy, placing this pathway firmly on the map in the pathophysiology of Parkinson's disease. PINK1 is a mitochondrial serine/threonine kinase encoded by the *PARK6* locus, while Parkin is cytosolic E3 ubiquitin ligase encoded by *PARK2*. PINK1 contains a canonical N-terminal mitochondrial targeting signal, followed by a transmembrane domain and a putative kinase domain at the C-terminus. Parkin contains a ubiquitin-like domain at the N-terminus followed by two RING domains (RING1 and RING2), an in-between domain and a third RING domain. This C-terminus section is termed the RING in-between RING (RBR) domain and contains the E3 ubiquitin ligase activity and HECT (homologous to the E6-AP carboxyl terminus)-like E3 activity. It has recently been shown that the dormant HECT-like domain of Parkin is activated by PINK1 and this activation is required for Parkin recruitment to mitochondria, and subsequent induction of mitophagy [107].

**Fig. 4:**
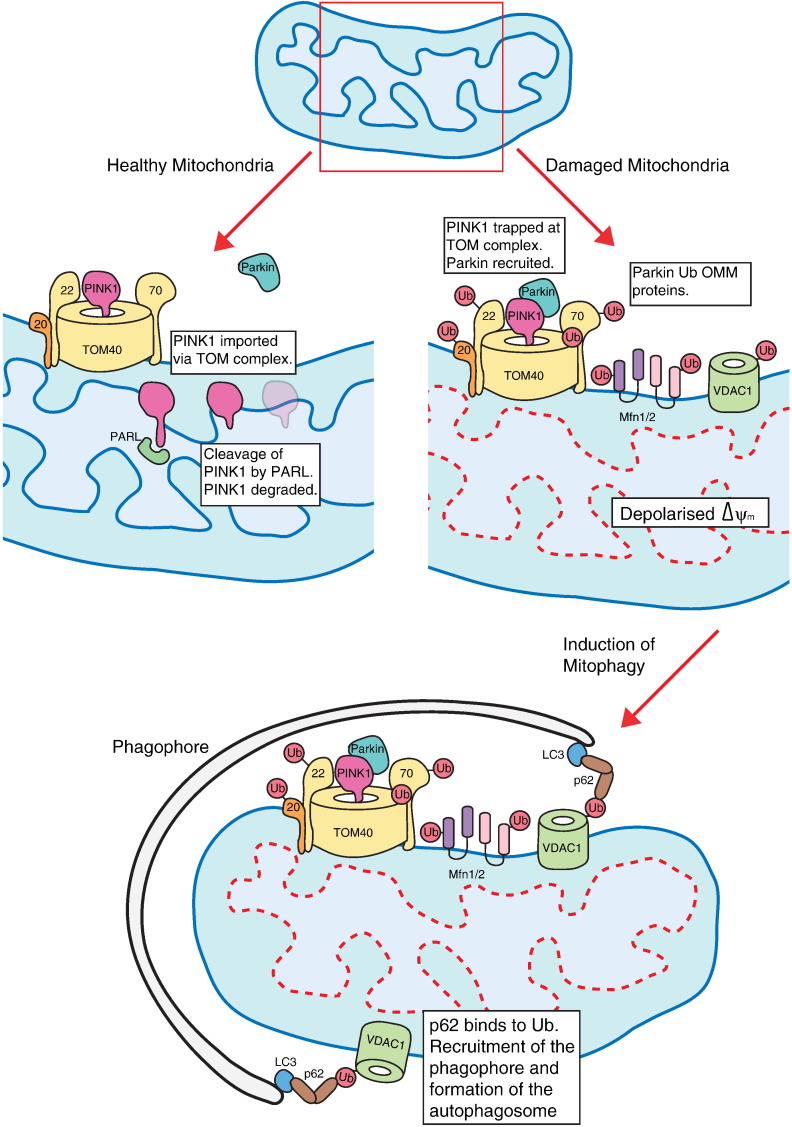
The mitophagy pathway. Under normal cell homeostasis PINK1 is imported in a membrane potential dependent manner. It localises to the IMM where it is cleaved by PARL. Loss of membrane potential (dashed red line) ensures PINK1 cannot be imported and is trapped at the TOM complex. This recruits the E3 ubiquitin ligase Parkin which ubiquitinates proteins on the cytoplasmic surface of the mitochondria. Ubiquitination of VDAC 1 recruits the adaptor protein p62/SQSTM1 which further binds LC3 and mediates recruitment of the phagophore. This mitochondrion is subsequently degraded by the autophagic pathway.

These proteins function in the same pathway with PINK1 acting upstream of Parkin. PINK1 is imported into the mitochondria in a ΔΨ_m_ dependent manner where it is cleaved by PARL in the IMM, restricting its expression (Fig. 4) [108–110]. This is a regulatory checkpoint in normal cell homeostasis. Upon dissipation of ΔΨ_m_, PINK1 import is inhibited and thus accumulates on the OMM, where it is trapped at the TOM complex [111]. PINK1 recruits Parkin and induces its ligase activity [112]. Ubiquitination of OMM proteins, Mfn1/2, components of the TOM complex (20, 40, 70) and VDAC1 complete the induction of mitophagy by the PINK1/Parkin pathway (Fig. 4) [74,113,114]. Site-specific mutation (C431) of Parkin has recently been shown to alter the ubiquitination patterns of proteins on the OMM [115]. Interestingly, it was also shown that there are no discernible sequence motifs common to proteins ubiquitinated by Parkin. This suggests that Parkin specificity is primarily determined by recruitment to the OMM and/or proximity to other proteins. Further supporting this, all modifications by Parkin to OMM proteins occur on the cytoplasmic face of the targeted protein [115]. Ubiquitination of the mitofusins appear to be of particular relevance as degradation of these proteins insures segregation of dysfunctional mitochondria, as there is no functional mechanism to tether adjacent mitochondria for fusion events.

The ubiquitin activity of Parkin is thought to be lysine-63 (K-63) mediated [74]. This polyubiquitination recruits p62, also called sequestosome 1 (SQSTM1) to the OMM. p62/SQSTM1 is thought to be an autophagy adaptor — although its role remains controversial. The protein has a K-63 ubiquitin binding domain in addition to an LC3-binding domain, thus it appears to have a role in recruiting autophagosomes to mitochondria [116]. Some reports detail a decrease in mitophagy upon knockdown of p62/SQSTM1 suggesting that it is limiting step in the process [117,118]. Conversely it has also been reported that p62/SQSTM1 mediates aggregation of damaged mitochondria, similar to the role it performs with polyubiquitinated proteins in the cytosol [119].

There are two sides to the process of autophagy/mitophagy. The first is a protective measure ensuring the removal of damaged organelles/proteins from the cell. However this process can be downregulated in disease states and in many cases can contribute to the pathogenesis of the disorder [83,84,120–122]. Although all the specifics of the process are not fully understood, the precise execution of autophagy and mitophagy pathways is clearly vital for cellular homeostasis.

## 5 Communications between the nucleus and mitochondria

### 5.1 Biogenesis and the control of mitochondrial capacity through transcription

Nuclear transcription controls mitochondrial function, ultimately coordinating the function and capacity of the organelle in response to intrinsic and extrinsic signals. The coordinated increase in mitochondrial mass and bioenergetic capacity is termed biogenesis. Activation of biogenesis can occur for many reasons, including alterations to the cell's redox state, nutrient deprivation, and (in muscle) exercise [123–125]. An array of transcription factors (TFs) activates and coordinates the expression of nuclear and mitochondrial genes with additional levels of control implemented through co-activators [126,127]. Nuclear respiratory factor 1 (NRF-1) and NRF-2 are the principal TFs associated with biogenesis. These TFs control the expression of key mitochondrial components involved in OXPHOS, haem biosynthesis, the function and regulation of mtDNA, and antioxidant defences [127]. Other TFs involved in the regulation of mitochondrial genes are the oestrogen related receptor (ERRα), the cAMP response element (CREB), and the Ying Yang 1 (YY-1) transcription factor. While ERRα regulates oxidative metabolism through its control of β-oxidation related genes [128], CREB and YY-1 are involved in the expression of ETC. protein components [129,130]. Although these transcription factors are directly responsible for the expression of mitochondrial proteins in the nucleus, higher level organisation through transcriptional co-activators integrates the TFs into the programme of biogenesis.

The peroxisome proliferator-activated receptor (PPAR) protein family includes one of the most studied regulators of biogenesis known as PPAR γ (gamma) co-activator 1α (PGC1α) [127]. PGC1α is considered a master regulator of biogenesis due to its crucial multidimensional role in the activation and regulation of transcription of nuclear encoded biogenesis factors [125]. It has two structural homologues known as PGC1β and PGC1 related co-activator (PRC). PGC1α and PGC1β exhibit a certain degree of complementarity in function and their expression is substantially increased under conditions requiring improved energy transduction through increased mitochondrial content [127]. Although PRC exerts control over a similar set of TFs to the other two homologues, it is thought to regulate biogenesis in response to different metabolic cues and stress signals [131]. Activation of PRC has been observed as a result of oxidant stress linked to inflammatory reactions and the premature induction of senescence [132]. This divergence in stress conditions capable of activating PRC suggests that it acts as a general regulator of adaptive responses to cellular dysfunction [132].

In addition to increasing the transcription of nuclear encoded mitochondrial genes, activation of biogenesis also increases the transcription of mtDNA encoded genes. The process of mitochondrial transcription requires the nuclear encoded RNA polymerase POLRMT, the transcription initiation factor TFB2M, the transcriptional stimulatory factor TFAM, and the termination factors MTERFs [15,133]. These factors cooperate in order to generate long polycistronic RNA precursor molecules from each strand of the plasmid like mtDNA. The polycistronic long RNA is subsequently processed to generate the mitochondrial rRNAs (mt-rRNA), mitochondrial tRNAs (mt-tRNA), and mitochondrial mRNAs (mt-mRNA) required for protein translation [134]. Apart from the rRNAs, tRNAs, and mRNAs, all other components of the mitochondrial translation machinery are nuclear encoded, however the process of translation in mitochondria is not well understood. Another feature of mitochondrial biogenesis is the potential for increased replication of mtDNA leading to an increase in mtDNA copy number. The replication of mtDNA is facilitated by the mitochondrial DNA polymerase gamma (POLG), which also exhibits repair activity [17,135]. Mutations in POLG, which lead to aberrant functionality are responsible for numerous disease phenotypes including Alper's syndrome and Progressive external ophthalmoplegia (PEO) [135,136]. A critical stage in the expression of mtDNA encoded proteins is their co-assembly with other subunits, including those that are nuclear encoded, into the OXPHOS complexes in the IMM. In order to facilitate assembly of the holoenzymes that constitute the OXPHOS machinery, a group of proteins known as ‘assembly factors’ orchestrate the correct formation of complexes, in a manner that is subject to a strict stoichiometric balance of protein subunits [137,138]. Problems arising with the assembly of complexes can lead to numerous mitochondrial diseases and highlight the importance of mitochondria to nucleus communication and interaction [139].

The modulation of mitochondrial capacity through the function of the PGC1 family and the process of biogenesis allows cells to respond to a variety of conditions requiring an increase in ATP transduction by utilising the highly efficient process of OXPHOS. Moreover, the effectiveness of the PGC1 family in dealing with conditions requiring increased mitochondrial capacity is highlighted by the therapeutic promise exhibited by induction of biogenesis through this system in models of ageing, neurodegeneration, and myopathies [125]. Nevertheless, the process of biogenesis is complex and relies on the effectiveness of a multitude of factors that will cooperate in order to bring about the efficient expression of proteins and assembly of macromolecular complexes in the mitochondria.

### 5.2 Retrograde signalling — when mitochondria talk back

The increased expression of nuclear encoded genes in response to altered mitochondrial bioenergetic state, compensating the effects of mitochondrial dysfunction or stress, is known as retrograde signalling (Fig. 2). Retrograde responses are not strictly considered part of mitochondrial QC systems, but are rather stress response pathways complementary to mitochondrial QC that activate mechanisms that tend to counter bioenergetic deficiencies caused by mitochondrial dysfunction [19]. Although a dedicated signalling pathway that performs this function has not yet been established in mammalian cells, studies in yeast have provided interesting observations on the workings of such a system. In yeast, perturbations to mitochondria such as the loss of mtDNA (i.e. petite or rho^0^ cells) lead to the activation of transcription by a factor comprised of the RTG1, RTG2, and RTG3 (RTG1–3) proteins [19]. Representative changes in metabolic function induced by retrograde signalling in yeast include the activation of the glyoxylate cycle and the upregulation of fatty acid β-oxidation [19]. These changes alter mitochondrial metabolism towards a greater variety of carbon sources, such as acetate, compensating for a reduction in capacity or lack of OXPHOS. Another interesting alteration to cell function in the yeast retrograde signalling model is the characteristic inactivation of TORC1, the yeast homologue of mTORC1 [140]. Active TOR signalling is a negative regulator of retrograde signalling components, while retrograde signalling activation and recruitment of the RTG factors inhibits TOR signals. Involvement of TOR signalling in the retrograde signalling pathway highlights the potential for the conservation of such a retrograde signalling system in higher eukaryotes. TOR signalling is itself highly conserved from yeast to humans and, although its sphere of activity may be slightly changed between species [141], its involvement may hint at the conservation of a retrograde signalling pathway.

In metazoans evidence for a retrograde signalling pathway has mainly been observed in the worm *C. elegans*, where its activation has been shown to extend lifespan [142,143]. In a study where life span was increased following RNAi mediated downregulation of COX4 (subunit 4 of cytochrome oxidase complex IV), there was a concomitant activation of the UPR^mt^ [144]. UBL-5 and DVE-1 were also required for the changes in nuclear transcription, suggesting interconnectedness between the many systems that facilitate mitochondrial to nucleus communication. Moreover, it supports a link between retrograde signalling and more traditional mitochondrial QC systems like the UPR^mt^. Evidence of retrograde signalling in metazoans has also started to surface from model systems such as the fly *Drosophila melanogaster* [145,146]. In mammalian model systems there are also some indications of retrograde signalling surfacing from studies in mice [147–149]. Results from these model systems demonstrate characteristic increases in life span. However, in the case of mammalian retrograde signalling, the pathway utilised for transducing the signal towards the nucleus and activating expression will be different from the RTG factors observed in yeast [19].

Although the exact pathway that may be utilised by a mammalian retrograde response still remains unknown, various evidence suggest that NFκB stress responses in mammalian cells resemble those observed as part of retrograde signalling in lower eukaryotes [150]. A recent study suggested that mitochondrial dysfunction caused by a specific mtDNA mutation, namely the A3243G base substitution principally associated with the mitochondrial encephalomyopathy lactic acidosis and stroke-like episodes (MELAS) clinical phenotype, leads to the activation of retrograde signalling in a mtDNA mutation cybrid model cell line [151]. Microarray data from cells containing mtDNA mutations suggests that the activation of systems relating to the observed retrograde response may be mediated through c-Jun/JNK pathway, as well as some involvement of RXRA. Both NFκB and c-Jun are rapid acting transcriptional regulators that do not require new protein synthesis in order to be activated. Such a rapid activation would be ideal in dealing with high priority stress signals such as those generated as part of mitochondrial retrograde signalling (Fig. 2).

### 5.3 The importance of genomes — nucleus and mtDNA compatibility

The ability of the mitochondrial genome to communicate and function synergistically with nuclear expression appears to also be determined by specific sequence alterations that categorise mtDNA in haplogroups. Extensively used to trace maternal phylogenetic lineages, mtDNA haplotypes also appear to have a considerable impact upon mitochondrial function [152]. Variations in mtDNA are specifically associated with disease phenotypes with specific sequence alterations in mitochondrial components that may generate measurable effects on bioenergetic and other functional aspects of the organelle, such as a decrease in respiratory function [153]. There are a multitude of pathogenic mutations reported in human mtDNA that associate with divergent clinical phenotypes [8], with a typical example being the A3243G mutation in the gene encoding mitochondrial tRNA^Leu^ [154]. Although the A3243G mutation is usually associated with the clinical phenotype known as MELAS it can also underpin the development of other clinical phenotypes. The multitude of pathological phenotypes that can arise in patients carrying the A3243G mutation stems from the fact that the mutation has a generic effect on the process of translation and protein production of mitochondrial components [8]. Nevertheless, it is clear that the same mutation can cause very different phenotypes between patients and an important variable that separates individual cases is the nuclear background. In fact substantial variation in mitochondrial function can also occur even when the sequence changes are not classically pathogenic. Elegant work by the Wallace lab has demonstrated how the balance of different haplotypes of mtDNA that are divergent due to polymorphisms in their DNA sequence, also known as heteroplasmy, can have a profound effect on cell physiology and can lead to substantial cognitive alterations in a mouse model [155]. Moreover, the different species of mtDNA in this study did not carry pathogenic mutations but innocuous alterations to the sequence, indicating that variation in mtDNA can impact upon more than just the expression of mitochondrial components. It is becoming clear that a certain amount of interaction between the nuclear and mitochondrial genomes occurs at a level not observable through our current understanding of biology. Yet mismatch between the two genomes results in observable effects upon the quality of mitochondria and cellular health. This suggests that the relationship between nuclear and mitochondrial genome backgrounds, which is most likely the result of co-evolutionary adaption between the two genomes, may potentially play a key role in health and disease.

## 6 Concluding remarks

Through this review we have explored some major aspects regarding the maintenance of mitochondrial fitness. Classical mitochondrial QC systems such as mitophagy and mitochondrial protein homeostasis, appear further enhanced and supported by mitochondrial stress response systems. All of these systems ultimately have a substantial effect on mitochondrial function, but may also be harnessed to correct mitochondrial dysfunction. Further exploration of these systems and better understanding of how they work, what activates, and what inhibits them, may ultimately provide us with methods to alleviate pathophysiological problems relating to aberrant mitochondrial function. To this effect the mitochondrion is now emerging as a target for therapeutic interventions that encompass small molecules, transcriptional regulation, and genetic manipulation, offering exciting opportunities to treat a diverse range of diseases.
